# The French Society of Geriatrics and Gerontology position paper on the concept of integration

**DOI:** 10.5334/ijic.1212

**Published:** 2014-03-27

**Authors:** Dominique Somme, Hélène Trouvé, Yves Passadori, Alain Corvez, Claude Jeandel, Marie-Aline Bloch, Geneviève Ruault, Olivier Dupont, Matthieu de Stampa

**Affiliations:** Department of Geriatrics, Université de Rennes 1, CHU de Rennes, La Tauvrais, rue de La Tauvrais, 35033 Rennes Cedex 9, France; Fondation Nationale de Gérontologie, 49, rue Mirabeau 75016, Paris, France; Department of Geriatrics, Centre Hospitalier Mulhouse, 87, avenue d'Altkirch, BP 1070, 68051 Mulhouse Cedex, France; ARS Languedoc-Roussillon, Parc-Club du Millénaire, 1025, rue Henri-Becquerel, CS 30001, 34067 Montpellier Cedex 2, France; Department of Geriatrics, Université de Montpellier 1, Centre Hospitalier Universitaire Centre Antonin-Balmès, Avenue Charles-Flahault, 34295 Montpellier Cedex, France; Research and Educational Innovation, École des Hautes Études en Santé Publique, Rennes, Sorbonne Paris Cité, équipe Management des Organisations en Santé (MOS), avenue du Professeur-Léon-Bernard, CS 74312-35043 Rennes Cedex, France; Délégation Générale de la SFGG, 60, rue des Cherchevets, 92150 Suresnes, France; Fondation Condé, Place Maurice-Versepuy, 60500 Chantilly; Université de Versailles-Saint-Quentin, Laboratoire Santé Environnement Vieillissement, EA 2506; Centre de Gérontologie, Hôpital Sainte-Périne, 49, rue Mirabeau, 75016 Paris, France

**Keywords:** position paper, definition, integration, geriatrics

## Abstract

**Introduction:**

The concept of integration, although dating from the 1990s, has only recently appeared in French public health policy. It must be linked with ‘coordination’, which is the base of most French public policies applied to geriatrics since the 1960s. Herein, we report the French Society of Geriatrics and Gerontology working group's findings according to three axes: definition of integration, objectives of this organisational approach and the means needed to achieve them.

**Discussion:**

Integration is a process that aims to overcome the fragmentation of services for vulnerable people. This process requires a multilevel approach, particularly concerning how to modify public policies and financing systems. Notably, all relevant levels need to develop shared processes, tools, resources, financing, interventions and action-reports on the latter. Integration must be accompanied by a local dedicated professional (the ‘pilot’). Results of recent experiments showed that it is possible to implement integrative dynamics in France.

## Introduction

The concept of integration of care and services, although dating from the 1990s, has only seen its real emergence worldwide since the 2000s, notably through the creation of dedicated international societies [1] (International Network for Integrated Care which became International Foundation for Integrated Care). It appeared later in France, with the National Alzheimer Plan 2008–2012 [2]. As society becomes more complex and tends to develop hyper-specialisations, it requires more integrated services able to respond better to the global needs of individuals and taking into account the current economic context.

In this setting, the French Society of Geriatrics and Gerontology wanted to clarify the concept of care and services integration for French gerontology by approaching the question from a theoretical (scientific literature) and a more direct practical perspective (knowledge obtained from gerontology actors, the reality in the field and recently conducted experiments). The society established an interdisciplinary working group (health, economics, laws and regulations) combining approaches to the concept to propose axes defining integration that could guide professionals and deciders concerning its application. This position paper, the fruit of 2 years of work by the group and six meetings, was written jointly by the authors and approved by the society.

## What is integration of care and services?

An analysis of the literature enabled us to collect several frequently cited definitions (Table 1) [1, 3, 4]. Several common points merit being underlined because they specify the most characteristic elements of the integration process.

## The integration target

The beneficiary is the least consensual of the definitions. When this target is identified, which is not always the case, it is characterised by a certain degree of vulnerability (associated with a handicap, the deterioration of general health with advancing age, etc.), necessitating recourse to different service providers.

## The integration levels

The extents of involvement of management services, health care and assistance delivery are always included in the definitions retained. However, the most comprehensive definitions also raise the roles of public funding and regulatory bodies.

## The sectors involved

Regardless of the definition, integration can only be considered a cross-sectional response. Although the definition can sometimes vary, the characteristic common to all of them is involvement of ‘different service systems (i.e., long-term care, professional education and development, home-aid services)’ [3].

Applying integration to a health care system in its entirety implies crossing the usual fragmentation lines between short- and long-term, primary and secondary health care systems, the different payment methods by act or activity and the social and public health sectors [5].

## Targeted objectives

The objectives raised in the definitions are often multiple. The most common characteristic is the subjective judgement of the elderly themselves of the system's character: integrated or not. Another frequent characteristic addresses continuity.

## Resources allocated and recommended methods

Different methods are often cited to establish integration. This pseudo-variability should not mislead the reader, because it is really quite simple to discern a central idea: integration depends on the possibility for different participants to share the resources, the interventions and the responsibility for the latter.

Therefore, we must exit the reasoning according each partner an exclusive isolated role for which this partner is only accountable. To do so requires that these participating institutions or organisations must first be interconnected, then act in unison (via cooperation or collaboration). The evaluation relies on the results of the service rendered to the target population in a setting of shared responsibility.

## A process more than a status

Reading these definitions allows us to realise the fundamentally ambitious and transforming character of integration. It is not a minor adaptation or negotiation of an arrangement, but a complete reorganisation of the system of services involved, from their definition and regulation to their delivery. It is easy to understand that such massive transformation cannot occur spontaneously or rapidly.

## How does integration differ from coordination?

In France, since the 1960s, numerous public policies have been based on the idea of coordination [6, 7]. Basically, these systems were not intended to transform the missions of existing organisations, but rather their interconnectivity. That is the essential difference with integration, which is less concerned with interconnectivity than the profound modification of the functioning of all the organisations. According to coordination logic, an actor is given a role to overcome the lack of coordination among the existing organisations or deficiency of services. Integration logic aims to modify the existing organisations so that, together, they find solutions to the fragmented services’ continuity ‘lived’ by the users. Thus, the latter can in no case be the ‘property’ or ‘task’ of a single organisation. Integration is, by nature, a collective project borne to fruition by partners. The concepts of integration and coordination are differentiated in Table 2. It is important not to consider integration the ideal and coordination useless. Coordination is often a necessary step or means favourable to achieving integration.

## Integration for the elderly: what is the goal? For which population?

The institutions and organisations do not have an obvious short-term interest in moving towards integration, which is a source of uncertainties and doubt. The main driving force comes from the inadequacies of the current response to the needs of the population.

An integrated system seeks to provide solutions to the consequences of fragmentation, including but not limited to: inappropriate hospitalisations, especially emergency admissions (by working before and after); repeated examinations evaluations and interventions; the difficulties of access to health care resources needed because of poor identification; ignorance of the overall assistance plan of action and absence of monitoring of care, with subsequent deterioration of the situation; the poor circulation of information among the different care levels (hospital–private practice, acute–chronic care and public–private); insufficient accounting of environmental factors in providing assistance to the individuals; the fatigue of caregivers faced with proposals whose interrelationship they do not grasp; insufficient consideration of the informal helpers in devising a collective response to individuals’ situations; undesired institutionalisation; poor transparency of the system, etc.

## How to achieve integration: what works and what should be avoided

The construction of integration requires reliance on interdependent mechanisms and tools [1, 8, 9]. These resources, essential to advancing integration, are the creation of area for cooperation, sharing the territory's access-to-services process; common evaluation and planning tools; and the existence of an information system. Relying on a human component favours successful integration; designating case managers for persons with complex living situations seems essential.

Having a pilot (local director of the management strategy for change) also seems to be a key factor in establishing services integration. Among available strategies, it seems necessary to avoid an exclusively top-down approach and opt for a dual top-down–bottom-up approach [9, 10]. Implementation of services integration should take into consideration the local context based on an organisational audit to understand the reality of the organisations and their professional practices to adapt the implementation strategy. In addition, the pilot overseeing the change must be in direct contact with the providers of care and support services.

## Conclusion

Integration of care and services aims at reducing the existing fragmentation of most western health care systems to make them more transparent for professionals and users. It is not an adaptation of the current structures but a complete reorganisation of care and assistance services, from regulation to delivery, necessitating the sharing of resources and interventions to respond to the users’ needs. Integration is a collective project/joint endeavour at the scale of a territory that requires being accompanied by a dedicated professional.

## Figures and Tables

**Table 1.**
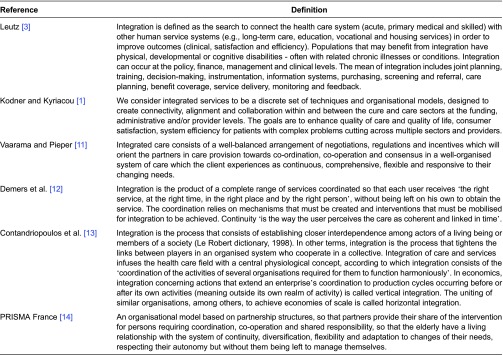
Definitions of integration

Note: PRISMA: Program of Research on the Integration of Services Maintaining Autonomy.

**Table 2.**
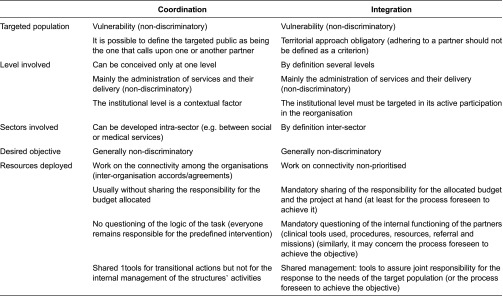
Comparisons of coordination and integration concepts as ways to organise systems

## About the Society

The French Geriatrics and Gerontology Society (SFGG) presentation (non-profit organisation existing since 1961), scholarly and scientific society uniting the skills of geriatrics and gerontology. The society goal is to study all issues related to gerontology and geriatric medicine, human ageing, senescence, longevity and promoting researches and works. The society takes on knowledge in all disciplines and regularly contributes to their dissemination. It brings together: The 16 French regional Geriatrics and/or Gerontology societies, the National Foundation of Gerontology (FNG) and a single specialised society: the French-Speaking Psychogeriatrics Society.

## Previous publication

This paper has previously been published in French in the following journals:

Gérontologie et Société

La Revue de Gériatrie

The original articles can be accessed here:

http://www.cairn.info/resume.php?ID_ARTICLE=GS_145_0201

http://www.revuedegeriatrie.fr/index.php#

Both journals have granted permission for this paper to be reproduced in English for publication in the IJIC.
